# Idiopathic Intracranial Hypertension Without Papilledema (IIHWOP) in Chronic Refractory Headache

**DOI:** 10.3389/fneur.2018.00503

**Published:** 2018-06-26

**Authors:** Valentina Favoni, Giulia Pierangeli, Francesco Toni, Luigi Cirillo, Chiara La Morgia, Samir Abu-Rumeileh, Monica Messia, Raffaele Agati, Pietro Cortelli, Sabina Cevoli

**Affiliations:** ^1^Unità Operativa Complessa Clinica Neurologica, IRCCS Institute of Neurological Sciences of Bologna, Bologna, Italy; ^2^Department of Biomedical and NeuroMotor Sciences, Alma Mater Studiorum–University of Bologna, Bologna, Italy; ^3^Neuroradiology Department, IRCCS Institute of Neurological Sciences of Bologna, Bologna, Italy

**Keywords:** chronic headache, refractory headache, idiopathic intracranial hypertension, MRI, lumbar puncture

## Abstract

**Background:** To determine the prevalence of Idiopathic intracranial hypertension without papilledema (IIHWOP) testing revised diagnostic criteria by Friedman in refractory chronic headache (CH) patients.

**Methods:** This is a prospective observational study. Each patient underwent ophthalmologic evaluation and Optical Coherence Tomography; brain magnetic resonance venography (MRV) and a lumbar puncture (LP) with opening pressure (OP) measurement. CSF withdrawal was performed in patients with CSF OP > 200 mmH20. IIHWOP was defined according Friedman's diagnostic criteria. Effect of CSF withdrawal was evaluated clinically in a 6-month follow-up and with a MRV study at 1 month.

**Results:** Forty-five consecutive patients were enrolled. Five were excluded due to protocol violations. Analyses were conducted in 40 patients (32 F, 8 M; mean age 49.4 ± 10.8). None had papilledema. Nine patients (22.5%) had OP greater than 200 mmH2O, two of them above 250 mmH2O. Two (5%) had neuroimaging findings suggestive of elevated intracranial pressure. One of them (2.5%) met the newly proposed diagnostic criteria by Friedman for IIHWOP. After CSF withdrawal seven (77.8%) of the nine patients improved. No changes in neuroimaging findings were found.

**Conclusions:** We found a low prevalence (2.5%) of IIHWOP in refractory CH patients according to current diagnostic criteria. In agreement with Friedman's criteria, our results confirm that a diagnosis of IIHWOP should be based on CSF OP and the combination of neuroradiological findings. However, where to set the CSF OP upper limit in IIHWOP needs further field testing. Although IIHWOP is a rare clinical condition, it should be considered and treated in refractory CH patients.

## Introduction

Chronic headache (CH) is a challenging clinical entity because management is complex and often unsatisfactory. A diagnosis of idiopathic intracranial hypertension without papilledema (IIHWOP) should be considered in patients with CH refractory to preventive therapy. Headache attributed to IIHWOP often mimics chronic migraine or chronic tension-type headache and the distinction from primary headaches can be clinically difficult. Lumbar puncture (LP) is mandatory to confirm diagnosis, but controversies exist regarding the CSF opening pressure (OP) cut-off value for the diagnosis of intracranial hypertension. The early accepted upper limit of normal CSF pressure was 200 mmH2O (1). Based on a long history of evidence (2, 3), recently, revised diagnostic criteria for pseudotumor cerebri syndrome by Friedman set the upper limit to 250 mmH2O and recognized the importance of MRI (4). According to these criteria, in the absence of papilledema or sixth nerve palsy, the diagnosis of IIHWOP can be suggested by the combination of elevated OP and the presence of at least three of the following neuroimaging findings: empty sella, distention of the perioptic subarachnoid space with or without a tortuous optic nerve, flattening of the posterior sclerae and transverse venous sinus stenosis (Table 1). Undoubtedly, the diagnosis of IIHWOP is mandatory to improve patient management. The aim of this study was to verify the prevalence of IIHWOP in chronic headache patients resistant to prophylactic therapies and to test revised diagnostic criteria by Friedman.

**Table 1:** Diagnostic criteria for pseudotumor cerebri syndrome (4).

**1. Required for diagnosis of pseudotumor cerebri syndrome^a^**
Papilloedema Normal neurologic examination except for cranial nerve abnormalities Neuroimaging: Normal brain parenchyma without evidence of hydrocephalus, mass, or structural lesion and no abnormal meningeal enhancement on MRI, with and without gadolinium, for typical patients (female and obese), and MRI, with and without gadolinium, and magnetic resonance venography for others; if MRI is unavailable or contraindicated, contrast-enhanced CT may be used Normal CSF composition Elevated lumbar puncture opening pressure (⩾250 mm CSF in adults and ⩾280 mm CSF in children [250 mm CSF if the child is not sedated and not obese]) in a properly performed lumbar puncture
**2. Diagnosis of pseudotumor cerebri syndrome without papilloedema**
In the absence of papilloedema, a diagnosis of pseudotumor cerebri syndrome can be made if B–E from above are satisfied, and in addition the patient has a unilateral or bilateral abducens nerve palsy. In the absence of papilloedema or sixth nerve palsy, a diagnosis of pseudotumor cerebri syndrome can be suggested but not made if B–E from above are satisfied, and in addition at least three of the following neuroimaging criteria are satisfied: Empty sella Flattening of the posterior aspect of the globe Distention of the perioptic subarachnoid space with or without a tortuous optic nerve Transverse venous sinus stenosis

a *A diagnosis of pseudotumor cerebri syndrome is definite if the patient fulfills criteria A–E. The diagnosis is considered probable if criteria A–D are met but the measured CSF pressure is lower than specified for a definite diagnosis*.

## Materials and methods

### Outcome measures

The primary endpoint of the study was to analyze the prevalence of IIHWOP in a series of consecutive refractory chronic headache patients.

The secondary endpoints were to test the revised diagnostic criteria by Friedman to detect IIHWOP and to verify the effect of CSF withdrawal on headache frequency and intensity.

### Standard protocol approvals and patient consents

The study was conducted in agreement with principles of good clinical practice and the study protocol was approved by the Local Ethics Committee of the local health service of Bologna, Italy (n. 12017/CE). All patients gave their written informed consent to study participation.

### Participants

Patients with refractory chronic migraine (CM) and Chronic Tension-type Headache (CTTH) with or without medication overuse referred to the tertiary Headache Centre of IRCCS Institute of Neurological Sciences of Bologna, Italy, were consecutively recruited from September 2013 to February 2016. Diagnosis of CM, CTTH and medication overuse were established according to the International Classification of Headache Disorders-3 beta version criteria (5). Refractoriness was defined as the failure of at least 3 trials of preventive therapies at adequate doses and at least one detoxification attempt in case of medication overuse. Inadequate response was defined as absence of reduction of migraine attack frequency or days by at least 50% after at least 3 months of therapy at a stable dose considered appropriate for migraine or tension type prevention according to accepted guidelines (6, 7). Exclusion criteria included the presence of papilledema on a routine fundoscopic examination, age <18 years, pregnancy and breast-feeding, secondary headaches, as well as serious ongoing physical or psychiatric illness.

### Study protocol

Each participant underwent: (1) a complete neurological and physical examination including height and weight measurements; (2) ophthalmologic evaluation with funduscopic examination and Optical Coherence Tomography (OCT) to rule out the presence of papilledema; (3) brain magnetic resonance imaging (MRI) including magnetic resonance venography (MRV) to detect neuroimaging features highly suggestive of idiopathic intracranial hypertension including transverse sinus stenosis, empty sella, flattening of the posterior aspect of the globe and distension of the perioptic subarachnoid space with or without a tortuous optic nerve; (4) an LP to measure CSF OP, which is the gold standard for the diagnosis of idiopathic intracranial hypertension. Prior to diagnostic procedures, we performed face-to-face structured interviews to gather detailed information about the clinical features of headache and associated symptoms including age at onset, days of headache per month, side and intensity of pain, years of chronic headache; presence, duration, and type of medication overuse; and potential related comordidities such as sleep apnea were ruled out. Headache diary data referring to 30 days before LP were collected as baseline evaluation.

### Optical coherence tomography (OCT)

Subjects underwent average and single sector (temporal, superior, nasal and inferior) RNFL thickness measurements by OCT (StratusOCT, software version 4.0.1; Carl Zeiss Meditec Inc, Dublin, CA, USA). We used the RNFL thickness 3.4 acquisition protocol, as previously described (8). Patients were compared with age- and sex-matched controls. For both groups we used a randomly selected eye for statistical analysis.

### MR imaging and MR venography

Each patient underwent an MRI morphological study of the brain, carried out with a 3T MR system (Signa 3T GE) with a standard head coil. All the examinations included T1 and T2 weighted sequences and MRV studies. MRV studies were executed with 2D time-of-flight sequence acquired in the coronal plane and with a 3D contrast-enhanced ultrafast gradient-echo angiographic sequence with elliptic centric ordering of k-space collection (Time Resolved Imaging of Contrast Kinetics-TRICKS). The sequence was acquired in the sagittal plane with covering the whole head. Venous phase correct time delay was calculated administering a small 2 ml test bolus of gadolinium chelates followed by 10 ml bolus of normal saline. The angiographic sequence was then acquired after the injection of a 30 ml bolus of gadolinium chelate contrast agent at a rate of 2 ml/s followed by 30 ml saline flush at 2 ml/s.

### Image processing and review

Source images from contrast-enhanced MRV (CE-MRV) of each patient were reviewed on a PACS viewing workstation by two neuroradiologists (F.T. and L.C.) who were blinded to the patient's clinical features. In addition to the sagittal source images it was also possible to review reformatted images of the volume using 1–2 mm thick section in the coronal and axial plane and 3D maximum intensity projection (MIP) reconstructions. Transverse sinus stenosis, empty sella, flattening of the posterior aspect of the globe and distension of the perioptic subarachnoid space with or without a tortuous optic nerve were defined as present or absent. The presence of at least three of four neuroimaging findings were needed to satisfied Friedman's criteria (4). Disagreements were resolved by consensus.

### Lumbar puncture

All LPs were performed as previously described, using a 20 gauge spinal needle and with the patient lying in the lateral decubitus position (9). CSF OP was measured by a standard spinal manometer calibrated in mmHg connected to the spinal needle via a three-way stopcock. All pressure values were multiplied by 13.56 (the Hg specific weight) for the conversion in mm H2O. We used the OP value of 250 mmH2O to define IIHWOP according to Friedman's criteria (4). According to previous findings that suggest that CSF withdrawal may result in a sustained remission of chronic migraine, we performed CSF withdrawal in patients with OP values above 200 mmH2O: intracranial pressure measurements were repeated every 2 mL of extracted CSF, up to about 100 mmH2O (9). In patients who showed an OP ≤ 200 mm H2O, the procedure was stopped after a 6 mL CSF withdrawal for routine analysis.

### Follow-up

A follow-up visit at 1 month was planned for all patients. Effect of CSF withdrawal was evaluated in patients with an OP > 200 mmH2O at 1, 3 and 6 months after LP. Moreover, the MRI study including MRV was repeated 1 month after CSF withdrawal to evaluate changes in neuroimaging findings. Patients recorded all headache attacks and drugs taken for headache during the whole study period on a clinical diary.

### Statistics

All data were analyzed using the SPSS software package (version 21- IBM Analytics). *T*-test or Mann-Whitney test, as appropriate, was used to compare continuous variables, while Chi-square test was adopted for categorical variables. Results were expressed by mean ± standard deviation, median with interquartile range (IQR) or percentage. The Spearman bivariate test was used to detect the strength of correlation between selected variables. Values of *p* < 0.05 were considered statistically significant. A Bonferroni correction was applied for multiple comparisons.

## Results

Forty-five patients were enrolled. Five patients were excluded due to protocol violations (one patient withdrew consent, two refused to undergo LP, one refused to undergo brain MRV and one refused to undergo both LP and MRV). Analyses were conducted in 40 patients (32 F, 8 M; mean age 49.4 ± 10.8; mean BMI 26.7 ± 6.4).

The diagnosis of headache was chronic migraine in 39 patients (97.5%) and chronic tension-type headache with concomitant episodic migraine in 1 patient. Mean chronification duration was 11.6 ± 9.9 years (range 1–32 years). At baseline mean headache frequency was 26.5 ± 6.5 days/month. Overall, 37 patients (92.5%) were medication overusers. The most commonly overused agents were triptans (*N* = 20; 54%) and non-steroidal anti-inflammatory drugs (*n* = 14; 37.8%). Fifteen patients (37.5%) overused two or more different compounds at a time (Table 2). Three patients complained of symptoms suggesting IIWHOP, such as tinnitus.

**Table 2:** Demographic and baseline clinical characteristics of the study sample.

		**TOTAL**
Sample	*N (%)*	40
Age	*mean ± SD*	49.4 ± 10.8
**SEX**		
Males	*N (%)*	8 (20.0)
Females	*N (%)*	32 (80.0)
**MARITAL STATUS**		
Single	*N (%)*	7 (17.5)
Married	*N (%)*	27 (62.5)
Separated/Divorced	*N (%)*	5 (12.5)
Widower	*N (%)*	1 (2.5)
Years of Education	*mean ± SD*	11.3 ± 3.5
**EMPLOYMENT**		
Unemployed	*N (%)*	3 (7.5)
Student	*N (%)*	1 (2.5)
Employee	*N (%)*	21 (52.5)
Housewife	*N (%)*	4 (10.0)
Retired	*N (%)*	6 (15.0)
Self-employed	*N (%)*	5 (12.5)
**BMI**		
<20	*N (%)*	2 (5.0)
20–25	*N (%)*	16 (40.0)
25–30	*N (%)*	14 (35.0)
>30	*N (%)*	8 (20.0)
**HEADACHE**		
Age at Headache Onset	*mean ± SD*	16.6 ± 8.3
Age of Headache Chronification	*mean ± SD*	37.8 ± 11.3
Duration of chronification in years	*mean ± SD*	11.6 ± 9.9
Headache frequency (days/month)	*mean ± SD*	28.1 ± 4.1
Frequency of medication intake (days/month)	*mean ± SD*	26.5 ± 6.9
**OVERUSED DRUGS**		
Triptans	*N (%)*	20 (50.0)
simple analgesics and/or NSAIDs	*N (%)*	21 (52.4)
Combination analgesics	*N (%)*	15 (37.5)

### Neurological examination

Neurological examination was unremarkable in all patients. None of them had unilateral or bilateral abducens nerve palsy.

### Ophthalmological examination and OCT

None of the patients had papilledema. All the patients underwent OCT. Four patients were excluded due to poor quality of OCT scan images. Thirty-six patients (29 F, 7 M; mean age 49.3 ± 10.8 years; range 22–67 years) were compared to 43 sex- and age-matched controls (32 F, 11 M; mean age 49.6 ± 12.1 years; range 23–68 years). The RNFL average thickness was within normal range in both groups and did not significantly differ between patients and controls (99.1 ± 8.9 μm in migraine patients vs 99.5 ± 13.0 μm in controls, *p* = 0.8). Moreover, there was no significant difference in the RNFL thickness in any of the optic nerve quadrants analyzed.

### LP

Two of forty patients (5%) had an OP above 250 mmH2O. Other seven (17.5%) had an OP between 200 and 250 mmH2O. All patients displayed normal CSF composition. We compared patients with OP < 200 mmH2O (Group 1) with those with OP > 200 mmH2O (Group 2). No statistical significant differences were found between group 1 and 2 regarding age, sex, years of education, duration of chronification (Table 3). Regarding BMI a statistical difference (*p* = 0.015) was found before Bonferroni correction. Two of the three patients with tinnitus have OP between 200 and 250 mmH2O.

**Table 3:** Comparisons of features of patients with OP < 200 mmH2O and patients with OP > 200 mmH2O (Group 1 and Group 2).

**Mean value ± *SD* Median (IQR) %**	**OP < 200 mmH2O (*N* = 31)**	**OP > 200 mmH2O (*N* = 9)**	***P*-values**	**Bonferroni adjusted *p*-values**
Age (years)	49±12	50±8	0.858	n.s.^*^
Female (%)	77.4	88.9	0.776	n.s^*^
BMI	25±5 24 (22–27)	32±7 34 (25–38)	0.015	n.s^*^
Opening pressure	159±28 163 (143–184)	245±51 224 (211–258)	<0.001	Significant^*^
Years of education	11±3 13 (8–13)	11±4 13 (8–13)	0.757	n.s.^*^
Duration of chronification in years	10±9 10 (3–15)	16±13 10 (5–28)	0.407	n.s.^*^

*n.s. not significant*.

* *values of p < 0.008 were considered statistically significant*.

### Neuroimaging criteria

**Figure 1:**
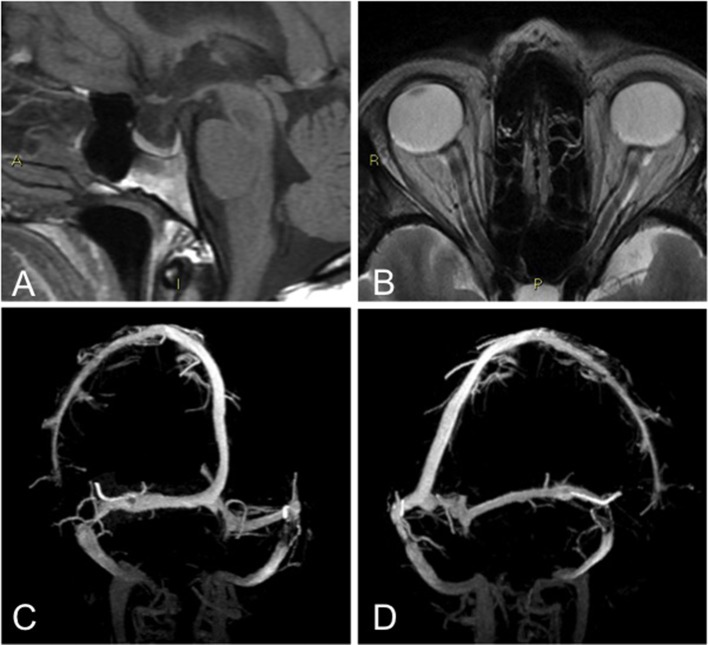
Patient that fulfilled Friedman's criteria: abnormally enlarged sella turcica that appeared partially empty **(A)**, Enlargement of perioptic subarachnoid spaces with faint flattening of the posterior aspect of the globe, on the left side **(B)**, bilateral dural sinus stenosis, at the distal portion of the transverse segment **(C,D)**.

All patients had normal brain parenchyma. Two of them (5%) had at least three of the four neuroimaging findings suggestive of elevated intracranial pressure as proposed by Friedman's criteria (Table 4). One of them had all the findings (Figure 1). The other had evidence of transverse sinus stenosis, empty sella, and distension of the perioptic subarachnoid space.

**Table 4:** Features of patients with CSF OP > 200 mmH2O.

**Patient**	**Sex; Age**	**BMI**	**Headache diagnosis**	**Duration of headache chronification headache (years)**	**Headache frequency (days/month)**	**Overused drugs and frequency of medication intake (days/month)**	**Number of neuroimaging findings (Friedman criteria)**	**CSF OP (mmH2O)**	**CSF withdrawal (ml)**	**Improvement after CSF withdrawal**	**Duration of improvement (follow-up)**
1	F;33	35.6	CM	2	25	Triptans (<10)	2	245	16	EM, Tinnitus	(Got pregnant)
2	F;46	35.2	CM	9	30	Combination analgesics; (30)	2	224	12	None	-
3	M;58	25.4	CM	10	30	Triptans, (30)	1	204	18	EM	3 months
4	F;58	25.4	CM	28	30	Combination analgesics; (30)	3	218	12	Reduced intensity of headache and drug intake	1 month
5	F;58	26.5	CTTH+EM	37	30	Triptans, (4)	2	204	8	None	-
6	F;46	43	CM	28	26	Triptans, (20)	0	224	8	EM	6 months
7	F;46	39.5	CM	2	30	NSAIDs; (10)	4	367	14	Reduced intensity of headache	1 month
8	F;51	22.1	CM	7	30	Triptans, (30)	0	245	20	EM	6 months
9	F;55	34.4	CM	17	20	Combination analgesics; (20)	0	272	26	Reduced intensity of headache and drug intake	1 month

*M, male; F, female; CM, chronic migraine; EM, episodic migraine; CTTH, chronic tension type headache. The presence of at least three of four neuroimaging findings were needed to satisfy Friedman's criteria*.

*Patients gave informed consent for the publication of the data contained in the table*.

### Prevalence of IIHWOP

One patient (2.5%) satisfied the diagnostic criteria for IIHWOP proposed by Friedman (Table 5). She had both OP > 250 mmH2O and all of the four neuroimaging features.

**Table 5:**
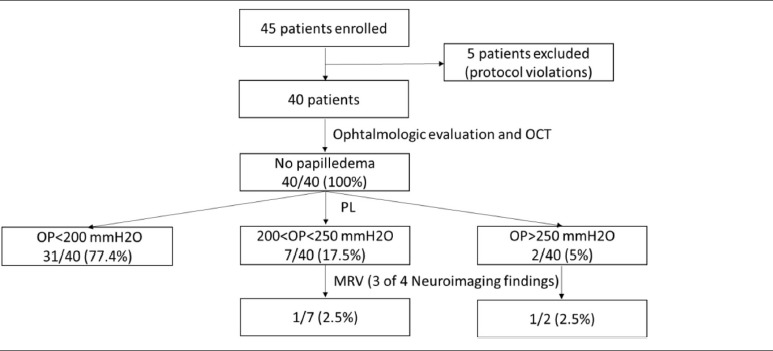
Flow chart of patients included in the study and results.

The other patient who had at least three of the four neuroimaging findings had OP > 200 mmH2O, but lower than 250 mmH2O. The other patient with OP > 250 mmH2O had none of neuroimaging criteria.

### Follow-up

At 1 month, considering the whole sample, 15 patients (37.5%) improved after LP, 23 (57.5%) had no benefit and 2 (5%) were lost at follow-up. Out of those that ameliorated, 8 had OP < 200 mmH2O, 5 had OP between 200 and 250 mmH2O, and 2 had OP > 250 mmH2O. Among them, there were no statistically significant differences regarding age (*p* = 0.694), sex (0.605), BMI (*p* = 0.072), years of education (p = 0.955) and duration of chronification (*p* = 0.897), even after Bonferroni correction between the subgroups of patients with OP < 200 mmH20 (*n* = 8) or those with *p*> 200 mmH20 (*n* = 7). Considering the 9 patients with OP >200 mmH2O, seven (77.8%) improved after withdrawal (Table 4). The two patients with OP > 250 mmH2O achieved an improvement of headache intensity and one of them a reduced frequency of medication intake, but no changes in the frequency of attacks. Among the five patients with OP ranging from 200 to 250 mmH2O, four experienced a return to episodic migraine and one decreased headache intensity and acute medications intake; one of them achieved also the recovery of tinnitus. A trial with acetazolamide was taken in account in patients with no contraindications to the use of this drug, and compliant to the treatment. All the 9 patients with OP>200 mmH2O repeated MRV 1 month after CSF withdrawal. No changes of neuroimaging findings were found.

At 3 months one of the two patients with OP > 250 mmH2O relapsed, none of them was treated with acetazolamide due to contraindications, and one was lost at follow-up. Among those with OP between 200 and 250 mmH2O, three patients still presented with an episodic migraine (two of them were treated with acetazolamide) and one patient was lost because she got pregnant. At 6 month of follow-up one relapsed to CM and two patients continued to have an episodic migraine (Table 4).

## Discussion

In our series, we found a low prevalence of IIHWOP in refractory CH patients. Only one of forty patients (2.5%) who had OP greater than 250 mmH2O and the neuroimaging finding suggestive of elevated intracranial pressure met the newly proposed diagnostic criteria by Friedman (4). Indeed, according to these criteria, CSF OP alone is not suitable for making a diagnosis and imaging has become an integral part of our diagnostic armamentarium for the diagnosis of IIHWOP. The upper limit of normal intracranial pressure is still debated (4, 10, 11). First of all, increased CSF OP may be due to several factors, including pain, anxiety, sedation, anesthesia, flexing of the neck and Valsalva maneuvers. In order to minimize false positive values a standardized procedure is essential with patients placed in a lateral decubitus position, as relaxed as possible, with legs stretched before pressure measurements. Moreover, CSF pressure monitoring demonstrated that intracranial pressure fluctuates during the day, ranging from normal to pathologic values. This implies that a single-spot opening measurement of CSF OP may fail to identify this condition, if the cut-off value is set at 250 mmH2O (12). However, although CSF pressure monitoring has a high accuracy in recognizing elevated intracranial pressure, it is an invasive procedure that is not practicable in routine use (12, 13). Moreover, the risk to overestimate the syndrome should be taken into account, if the cut-off value is set at 200 mmH2O. Asymptomatic OP greater than 200 mmH2O was found in 11% of neurological patients, probably similar to the general population (10). Previous studies in headache patients reported OP > 250 mmH2O ranging from 5 to 55.5% and OP > 200 mmH2O ranging from 10 to 100% (Table 6) (9, 12–17). These heterogeneous results may be mainly due to different methods and patient selection. Moreover, limited data are available in CH patients, especially those refractory to treatments. In our series, we found OP > 250 mmH2O and OP > 200 mmH2O in 5 and 22.5% respectively. Our results are significantly lower than those recently reported in a series of 44 unresponsive chronic migraine patients, where OP > 250 mmH2O and OP > 200 mmH2O were found in 43.2 and 86.4% respectively (9). In this latter study MRV was used for selecting patients with transverse sinus stenosis, however the difference in outcome cannot be easily explained by different patient selection criteria (18).

**Table 6:** Summary of previous studies performing CSF pressure measurement in headache patients.

**Author (Year)**	**Headache patients that underwent LP (n)**	**Diagnosis of headache**	**Method of CSF pressure measurement**	**CSF pressure >200 mmH2O**	**CSF pressure >250 mmH2O**
(14)	85	refractory CM	single LP, 20G needle	12/85 (14.1%)	11/85 (12.9%)
(13)	10	refractory CM, NDPH, CTTH	CSF monitoring, 14G needle	9/9 (100%)	5/9 (55.5%)
(15)	28	Migraine	single LP, 20G needle	19/28 (67.8%)	NA
(16)	60	CM	single LP, 22G needle	6/60 (10%)	3/60 (5%)
(17)	13	CTTH	single LP, 20G needle	9/13 (69.2%)	6/13 (46.1%)
(12)	48	CM, CTTH	1h-CSF monitoring, 20-22G needle	18/48 (37.5%)	NA
(9)	44	refractory CM	single LP, 20G needle	38/44 (86.4%)	19/44 (43.2%)

*CM, chronic migraine; CTTH, chronic tension type headache; LP, lumbar puncture; NDPH, New Daily Persistent Headache*.

To our knowledge, our study is the first one that focused on the combination of neuroradiological findings and OP in refractory CH patients to test IIHWOP criteria. Between the two patients (5%) that satisfied neuroimaging criteria, both have OP higher than 200 mmH2O, but only one higher than 250 mmH2O. On the contrary, considering CSF OP > 250 mmH2O alone, the prevalence of IIHWOP may rise to 5%. However, between the two patients with OP greater than 250 mmH2O, one failed to meet diagnostic criteria due to the absence of neuroimaging findings. Despite the effort to use a standardized procedure, we cannot exclude that the OP found in this patient was a false positive value. Consequently, using CSF OP alone, the risk is to overestimate a diagnosis. These findings confirm the need to combine OP and neuradiological criteria to make the diagnosis.

Moreover, we confirm that neuroimaging features persist after CSF withdrawal (19). This is in contrast with recent evidence showing the reversibility of stenosis after intracranial pressure normalization (20, 21). The difference in outcome can be related to different timing of MRV after withdrawal and to the hypothesis that transverse sinus stenosis is only one of the contributing factors involved in IIHWOP (19).

Our results confirm that CSF withdrawal may have clinical effects on headache in CH patients, with long-term effect (9, 12, 16, 22). A proportion of 77.8% of our refractory patients with OP > 200 mmH2O reported improvement of headache frequency and/or intensity until up to 6 months after withdrawal. Only partial improvement was reported by patients with OP > 250 mmH2O, but the number is too small to make certain assumptions. Moreover, a placebo effect could not be excluded, considering that 25.8% of patients with OP < 200 mmH2O improved after LP. However, we are pretty certain that clinical features did not differ between patients with OP>200 mmH2O and patients with OP < 200 mmH2O. Interestingly, it appears that there was a trend toward statistical significance with the OP and BMI. Patients with higher BMI are trending toward higher OP. Based on a previous meta-analysis of observational studies that suggests an association between migraine and obesity (23), we suppose that refractory headache may be related with BMI and OP. Moreover, we can argue that patients with OP > 200 mmH2O may potentially be a subgroup of those with “pre IIH” as post CSF removal, 7 out of the 9 had improvement of symptoms.

In addition, we suggest that funduscopic evaluation is sufficient and mandatory to exclude papilledema in refractory CH patients, considering that none of our patients with negative funduscopic exam had OCT sign of papilledema. As a corollary, in contrast to a previous report in chronic migraine patients, all our refractory patients had RNFL thickness within normal range, supporting the hypothesis that long duration of headache does not affect the eye (24).

The study was limited by the relatively small sample size to compare patients with OP > 250 mmH2O with those with OP > 200 mmH2O and to evaluate the effect of CSF withdrawal. However, for feasibility reasons we did not recruit further patients. Moreover the effect of CSF withdrawal should be investigated by mean of a randomized control study. We think that a future multicenter study with a larger number of refractory CH patients is necessary in order to reach meaningful results and better define the characteristics of patients with OP > 250 mmH2O.

## Conclusion

IIHWOP is a rare clinical condition that should be considered in patients with chronic headache refractory to medical treatment. Our results confirm that the combination of neuroradiological findings (transverse sinus stenosis, empty sella, flattening of the posterior aspect of the globe and distention of the perioptic subarachnoid space) support the presence of increased intracranial pressure. Determination of CSF opening pressure remains mandatory when brain RM finding suggest pseudotumor cerebri and may result in headache improvement. We agree with Friedman's diagnostic criteria that a diagnosis of IIHWOP should be based on the CSF OP and the combination of neuroradiological findings.

However, based on our results, where to set the CSF OP upper limit in IIHWOP, which may be a milder form of pseudotumor cerebri, remains an open question. We suggest that diagnostic criteria should be tested in other studies in different centers in order to validate or revise it.

## Author contributions

VF, FT, LC, CLM, and SC: conceived and designed of the study; VF: supervised the study; VF, GP, FT, LC, CLM, MM, RA, and SC: acquired and interpreted the data; CLM and SA-R: performed the statistical analysis; VF, FT, and SC: drafted the manuscript; GP and PC: substantially contributed to conception and design of the study and critically revised the manuscript. All authors contributed to manuscript revision, read and approved the submitted version.

### Conflict of interest statement

PC has received honoraria for speaking engagements or consulting activities with Allergan Italia, AbbVie srl, Chiesi Farmaceutici, Teva, UCB Pharma S.p.A, Zambon. The remaining authors declare that the research was conducted in the absence of any commercial or financial relationships that could be construed as a potential conflict of interest.
